# Pattern of Road Traffic Injuries: A Study From Western Maharashtra

**DOI:** 10.4103/0970-0218.39248

**Published:** 2008-01

**Authors:** Supriya Satish Patil, RV Kakade, PM Durgawale, SV Kakade

**Affiliations:** Department of Community Medicine, Krishna Institute of Medical Sciences Deemed University, Karad, Maharashtra, India

## Introduction

Injuries are increasingly recognized as a global public health epidemic. Around the world, almost 16,000 people die everyday from all types of injuries. Injuries represent 12% of the global burden of disease, the third most important cause of overall mortality and the main cause of death among 1-40 year age groups.(1) The category of injuries worldwide is dominated by those incurred in road crashes. According to WHO data, deaths from road traffic injuries account for around 25% of all deaths from injury.(1)

India has one of the highest road accident rates in the world. There has been a steady rise in the casualties in road accidents in the country and their proportions in total deaths due to all accident have also increased considerably in the past. In India, nearly 80,000 get killed and 340,000 are injured every year in about 300,000 accidents on road network of just 22,00,000 km^2^. There is an accident every minute and death every 8 min. Significant variations also arise between different states of India.(4)

## Materials and Methods

This study was conducted in Krishna Hospital and Medical Research Centre (KH and MRC), by the Department of Preventive and Social Medicine from 1 May 2003 to 30 April 2004. This hospital is attached to Krishna Institute of Medical Sciences Deemed University, Karad, Maharashtra. All road traffic injury cases admitted during above one-year period were studied and analyzed. The patients admitted with delayed complications of road traffic injuries were not included in the study.

Data were collected everyday by the candidate either in the casualty or in the wards of KH and MRC. A pretested proforma specially designed for this purpose was used for interviewing the study subjects. Where condition of victims did not warrant the interview, the relatives or attendants were interviewed. During the interview, purpose of study was explained to each respondent. Case-sheets of the victims were referred for cross-checking. The information collected consisted of personal identification data, history of road traffic injuries, which included human and environmental risk factors and clinical history and examination, the type and severity of injury suffered by the victims was graded using the “trauma index”. The treatment given and the outcome were also recorded for each case.

For the purpose of study definitions used were:

Road traffic accident (RTA) - A collision involving at least one vehicle in motion on a public or private road that results in at least one person being injured or killed.

Road traffic crash (RTC) - A collision or incident that may or may not lead to injury, occurring on a public road and involving at least one moving vehicle.

Road traffic injuries (RTIs) - Fatal or non-fatal injuries incurred as a result of road traffic crash.

## Results

A total of 350 cases of road traffic injures from 301-road traffic accidents were reported at Krishna Hospital and Medical Research Centre, Karad during the study period.

There were 288 (82.3%) male and 62 (17.7%) female casualties. The average age of victims was 32.5 years. The highest number 103 (29.4%) victims were between 20 and 29 years of age. There were 37 (10.5%) children below 14 years of age and 21 (6%) victims were aged 60 and above. In the present study, considering all age groups, males were more commonly involved than females with the ratio of 4.6:1.

Of 174 (49.7%) occupants, motorized two-wheeler occupants were highest in number, i.e. 61 (35%) followed by occupants of four wheelers - 45 (25.9%), other occupants were from truck - 27 (15.5%), three wheelers - 19 (10.9%), tractors - 10 (5.7%), tempos - 4 (2.3%), bullock carts - 4 (2.3%), bicycles - 3 (1.7%) and bus - 1 (0.6%).

Pedestrians were 47 (13.4%). Fifteen (31.9%) were injured by motorized two wheelers; four wheelers caused injury to 14 (29.8%) pedestrians. Tractor and trucks were involved in six (12.8%) and five (10.6%) pedestrians.

A total of 129 drivers were involved. Among the drivers of different types of vehicles, there were 79 (61.2%) motorized two-wheeler drivers and 26 (20.1%) bicyclists. Truck drivers were three (7%), whereas bullock cart, three-wheeler and tempo drivers were two (1.6%) each. Tractor drivers were three (2.3%), four-wheeler drivers were five (3.9%) and bus drivers were one (0.8%).

Of 129 drivers involved, 28 (21.7%) were bicyclists and bullock cart drivers who did not require a license. From the remaining 101 (78.3%) drivers of different motor vehicles, one had learner's driving license and 38 (29.5%) had no valid license. Those who had no license, 35 (92.1%) were driving motorized two wheelers.

In the present study, out of 129 vehicle drivers, 38 (29.5%) were under influence of alcohol. We have obtained history of alcohol consumption from relatives or patients themselves (by the smell of alcohol).

Out of 64 four wheelers, 48 (75%) were overcrowded (occupancy more than permitted capacity). There is a significant association between type of vehicle and overcrowding (χ^2^ = 110.888; df = 8; *P* < 0.001). Overcrowding was there in 38 (92.7%) trucks, 13 (68.4%) tractors, 6 (85.7%) tempos, 18 (78.3%) three wheelers, 41 (26.5%) motorized two wheelers, 6 (20.7%) bicycles and 5 (71.4%) bullock carts. Out of 48 overcrowded four wheelers, 22 (45.8%) were Wadap jeeps and out of 18 three wheelers, 11 (61.1%) were Wadap rickshaws.

A total of 190 fractures were noted among the victims. The commonest site of fracture was lower limb 88 (46.3%) followed by upper limb 47 (24.7%) and skull 25 (13.2%). Other sites were spine 12 (6.3%), ribs 11 (5.8%) and pelvis 7(3.7%). Fatality rate in our study was 3 (0.8%).

The severity of injury according to category of road users showed that two (1.6%) drivers, five (2.9%) vehicle occupants and one (2.1%) pedestrian have severe injuries. There is no significant association between severity of injuries and category of road users (χ^2^ = 7.937; df = 4; *P* > 0.05).

## Discussion

In the present study, the highest number of victims (29.4%) was between the age group of 20-29 years. The people of the third decade are more commonly involved in road traffic injuries. In the present study, 64.9% of the victims were between 15 and 44 years age group. Similar observation was reported by WHO in *The Injury Chartbook*.(2) This shows that the people of the most active and productive age groups are involved in road traffic injures, which add a serious economic loss to the community. The present study shows that below the age of 14 and above the age of 60 years, the proportion of victims was low. Corresponding findings were reported by Jha *et al*.(3)

The male-to-female ratio was 4.6:1. It was observed that 80% of the victims were males.(5) The gender difference is probably related to both exposure and risk taking behaviour.

In this study, the vehicle occupants constituted the highest (49.7%). Maximum (31.9%) pedestrians were injured by motorized two wheelers. Corresponding results were reported by Jha *et al*.(3) Among the drivers, motorized two-wheeler drivers (61.2%) were commonly involved. This could be due to higher speed, which can be achieved over short distance and less stability of the vehicle.

This study found that 29.5% drivers of different vehicles were without driving license. The reason may be the easy accessibility of the vehicles and casual attitude of drivers towards obtaining license. Being a rural area, lack of awareness and inadequate enforcement of existing laws could be the other reason. When they were interviewed, they mentioned that they would be applying for and obtaining the license in future.

In the present study, 29.5% drivers involved had consumed alcohol.

Among fractures, the present study found that lower limbs (46.3%) were the commonest site for fracture, followed by fracture of upper limbs (24.7%) and skull (13.2%). Similar findings were reported by Jha *et al*.(3) Studies have reported that the highest number of fractures were in upper limbs followed by lower limbs and facial bones. However, their study was confined to only motorcycle accidents, whereas the present study takes into account road traffic injuries due to all types of vehicles. The extremities are commonly involved due to direct trauma of the vehicle or due to fall. The extremities are more vulnerable to injuries especially in motorcyclists because they are unprotected.

Several human and environmental risk factors such as age, alcoholism, without driving license, type of vehicle, etc. were found associated in occurrence of road traffic injuries. If we control these factors appropriately, mortality and morbidity can be prevented.
